# TreeHelper: A Wood Transport Authorization and Monitoring System

**DOI:** 10.3390/s25216713

**Published:** 2025-11-03

**Authors:** Alexandru-Mihai Zvîncă, Sebastian-Ioan Petruc, Razvan Bogdan, Marius Marcu, Mircea Popa

**Affiliations:** Department of Automation and Computing, Politehnica University Timisoara, 300006 Timisoara, Romania; alexandru.zvinca@student.upt.ro (A.-M.Z.); sebastian.petruc@upt.ro (S.-I.P.); razvan.bogdan@cs.upt.ro (R.B.); marius.marcu@cs.upt.ro (M.M.)

**Keywords:** IoT, deforestation monitoring, TPU, cloud, RaspberryPi, 4G, EdgeAI

## Abstract

This paper proposes TreeHelper, an IoT solution that aims to improve authorization and monitoring practices, in order to help authorities act faster and save essential elements of the environment. It is composed of two important parts: a web platform and an edge AI device placed on the routes of tree logging trucks. The web platform is built using Spring Boot for the backend, React for the frontend and PostgreSQL as the database. It allows transporters to request wood transport authorizations in a straight-forward manner, while giving authorities the chance to review and decide upon these requests. The smart monitoring device consists of a Raspberry Pi for processing, a camera for capturing live video, a Coral USB Accelerator in order to accelerate model inference and a SIM7600 4G HAT for communication and GPS data acquisition. The model used is YOLOv11n and it is trained on a custom dataset of tree logging truck images. Model inference is run on the frames of the live camera feed and, if a truck is detected, the frame is sent to a cloud ALPR service in order to extract the license plate number. Then, using the 4G connection, the license plate number is sent to the backend and a check for an associated authorization is performed. If nothing is found, the authorities are alerted through an SMS message containing the license plate number and the GPS coordinates, so they can act accordingly. Edge TPU acceleration approximately doubles TreeHelper’s throughput (from around 5 FPS average to above 10 FPS) and halves its mean inference latency (from around 200 ms average to under 100 ms) compared with CPU-only execution. It also improves p95 latency and lowers CPU temperature. The YOLOv11n model, trained on 1752 images, delivers high validation performance (precision = 0.948; recall = 0.944; strong mAP: mAP50 = 0.967; mAP50-95 = 0.668), allowing for real-time monitoring.

## 1. Introduction

### 1.1. Context and Motivation

The latest data shows that 31% (4.1 billion ha) of the world land surface is covered by forests, with 54% of the world’s forests in only five countries—Brazil, Canada, China, Russia and the United States of America. Although the rate of deforestation is decreasing [1,2], the overall situation is still alarming. Since 1990, 420 million ha of forests have been lost through deforestation.

**Figure 1:**
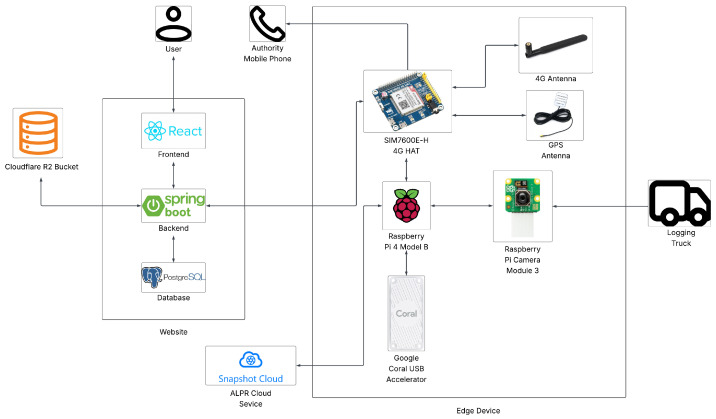
System architecture.

In recent years, approximately 4 billion m^3^ of roundwood has been removed from forests annually. Half of this quantity is being used as fuel, while most of the other half is used as material for industrial production. These numbers are at an all-time high. Using wood as fuel is most common in lower income communities, wood being the most affordable and reliable fuel source. A total of 82% of wood fuel is produced and used in Africa, Asia and South America [2]. Furthermore, USDA projections show a clear increase in general roundwood production, with a range from 6% to 32% between 2022 and 2050. The same projections also describe a likely increase in industrial roundwood demand and a small decrease in roundwood for fuel [2]. For more context regarding industrial usage, Figure 1 breaks down the share of global forest product exports.

A particular study case is Romania. Recent metrics show that Romania has 698,162 ha of land with forests, equal to 29% of the total land area of the country. It ranks 20th in the European Union and is situated under the EU average of 38.6% of forested area [3,4]. The country is well known for its ancient forests. According to the National Forests Inventory (NFI) 2013–2018, 7.6% of Romanian forests are more than 120 years old and approximately 1% are old-growth (virgin or quasi-virgin), meaning from more than 160 years old up to 300–400 years old for the most common European tree species; the removal of these trees contributes to economic problems and flooding [5,6,7].

Unfortunately, deforestation and illegal logging are real problems in Romania, affecting wildlife, communities and the healthy legal practice of wood gathering. National Forests Inventory data shows a rise in illegal wood exploitation. Approximately 20 million m^3^ of wood is gathered illegally annually, various recent analyzes linking this phenomenon with patterns of deforestation in Romania [8,9,10]. Authorities have only been able to find 1% of this quantity and identify the culprits, a dramatically small number. The three counties with the highest rate of illegal activity are Maramureș, Bistrița and Sibiu.

The facts already mentioned clearly highlight the need to save forests and ensure that illegal and unhealthy practices regarding wood exploitation are reduced as much as possible. The Food and Agriculture Organization of the United Nations (FAO) categorizes technological innovation as one of the five essential pillars of enhancing the potential of forests, thus digitalization of administrative measures is put under the spotlight [2].

Taking into account all of the above, we have decided to develop a prototype for an authorization and monitoring system, paving the way for a large-scale implementation and integration with current solutions that could impact the whole silviculture sector in a positive way. The system is made up of a web page that allows users to register and request wood transport authorizations; authorities can approve or deny these requests on a case-by-case basis. Edge AI devices will be situated on roads that are commonly used for wood transport and will have a camera, which, with the help of an artificial intelligence model, will be able to recognize logging trucks and their license plates. This approach has been demonstrated as feasible in [11]. Using a cellular connection, the license plate number is verified and, if there is no authorization, the authorities will be notified of illegal wood transports via SMS.

Given a roadside camera stream and 4G connectivity, the system must

1. Continuously capture frames and maintain a GPS fix on the device.
2. Detect logging trucks locally and in real time using a compact YOLOv11n model.
3. Extract the vehicle license plate by sending a frame to a cloud automatic license plate recognizer service to obtain a returned string.
4. Query the backend authorization endpoint with the plate string in order to determine whether a valid transport authorization exists.
5. Trigger an alert when no authorization is found, notifying the competent authorities via SMS, including the plate and GPS coordinates in the message, for timely intervention.
6. Apply safeguards for reliability to minimize false or duplicate alerts.

Success is defined by continuous real-time operation on the edge and accurate end-to-end decisions based on transport authorizations for passing trucks in an environment suitable for the use case.

### 1.2. State of the Art

The digitalization of forest protection measures has been studied through research papers and has been implemented through various products, both at a public sector level and at a private sector level.

SUMAL 1.0 (Electronic Timber Tracking System) was launched by the Romanian Government in 2008 and was the first system of this kind implemented in Romania, a milestone for digitalization. The software was intended to be a tool that would eliminate physical bureaucracy, standardize processes and host all the relevant wood tracking data in one place. Its functionalities included the generation of administrative documents for both authorities and silviculture companies, computation of relevant values and statistics and overall verification and monitoring of the whole process of wood exploitation. SUMAL 1.0 was a desktop software program [12].

In 2016, a complementary system was added, called “Inspectorul Pădurii” (The Forest Inspector). Here, individuals can manually input the license plate number and other information in order to check if an authorization exists for a specific timber transportation activity. Furthermore, the system allows the user to check all the relevant documents regarding the transport. The Forest Inspector comes as both a website and a mobile application and is connected to SUMAL, instantly verifying all the given information. If the users receive a message that the transport does not have valid credentials, they need to call the emergency number in order for the authorities to take immediate action [10].

The second iteration of SUMAL, SUMAL 2.0, was launched in 2020. It came with upgraded technologies and a change of paradigm. One of the biggest additions was the GP-based monitoring, all the wood transportation trucks being now required to have tracking devices installed. It also came with a lot more data regarding legal issues and documents and with a new and modern UI. The Forest Inspector has also been integrated with SUMAL 2.0. It is made up of three main components: a web platform, mobile applications and a central hardware–software unit in the custody of the Special Telecommunication Service. The website allows users to register and add all relevant and required information about their activities. Similar to SUMAL 1.0, it helps with document generation, computations and can showcase all public documents. The mobile applications are split in order to clearly differentiate between specific aspects of the whole wood exploitation legal framework. Each application is designed for a different use case. SUMAL 2.0 is still in use at the time of writing this paper, and The Forest Inspector has also been integrated with the system [11].

In 2021, Vodafone Romania proposed an IoT-oriented solution to combat illegal logging. The project is called “Romania’s First Smart Forest” and takes the form of a network of solar powered devices used for acoustic monitoring, similar to the ones in [13]. They are installed on the canopy of the forests and continuously collect sounds from the surroundings, sending them to an analyzing application in real time. The application uses artificial intelligence to detect sounds that could give signs of illegal logging, for example the sound of chainsaws. As a last step, forest rangers receive all the data on their phones, including the captured sounds and GPS data, so a quick intervention can be made. The transmission of data is performed with the help of the Vodafone cellular network [13].

Rainforest Connection (RFCx) uses a system very similar to the one Vodafone is implementing in Romania; it is based on the same principles. Various other systems combining Internet of Things and ML technologies confirm that the approach is highly functional [13,14]. Rainforest Connection furthers the scope by using artificial intelligence analysis of sounds to categorize species of animals, gather data about the ecosystem and stop animal poaching. So far, the system is being used in 37 countries and monitors 736,200 ha of land. Another acoustic-based solution that has been implemented is GreenSoal, which used IoT and ML to detect suspicious sounds. It uses a Raspberry Pi Model 3 as the central controller and an Arduino Uno as the secondary node. It is solar powered and also has digital microphones and GSM modules (nRF24L01+). The sound recognition module utilizes Fast Fourier Transform (FFT) and spectrogram analysis to convert audio data to visual time-frequency data. These samples are then used to train a neural network that has been shown to achieve over 93% accuracy in classification [14].

XyloTron takes a different approach to this issue: it analyzes the structure of the wooden material using AI (based on ImageNet) [15]. It is a portable system that does not require an Internet connection. The specimen classification accuracy is approximately 97.7%, and it takes microscopic images as inputs. Thus, it is used to identify unauthorized timber usage in order to stop such products entering into supply chains [15].

The combination of machine learning for vehicle recognition and optical character recognition (OCR) for automated license plate recognition (ALPR) is quite common in urban applications with edge-AI and YOLO-based technologies, improving their applicability in real-time scenarios [12,13,16,17]. Speed cameras, smart parking lots and other smart city solutions use these concepts in order to automate many trivial and vital processes. Usually, these systems have access to the Internet either via WiFi or Ethernet and can facilitate real-time backend and database actions.

Anmol Singhal and Navya Singhal introduced PatrolVision, an ALPR system similar to the systems found in [18,19,20,21,22] and designed for urban authority vehicle patrolling use cases, which tries to tackle unconstrained environments where license plates can very often be distorted. This study provides a great step forward towards automatic traffic law enforcement. The system uses a YOLO (You Only Look Once)-based ML model trained on 16,910 images captured under varying light conditions and from different angles and reaches 86% precision on the detection of license plates with 67% full character matching. PatrolVision is an end-to-end system with an automated pipeline of four tasks: license plate detection (LPD), license plate segmentation (LPS), character recognition (CR) and sequence reconstruction. Furthermore, the paper emphasizes the feasibility of using such systems on edge devices, reaching real-time performance on Nvidia Jetson TX2 [18].

The Forest Guard project, developed by Patrick Neicu, is a well-rounded initiative to combat illegal logging. The approach is composed of three big segments. Firstly, license plate detection is obtained with the use of YOLOv8 and an OCR API for text extraction, similar in principle to [16,23,24,25]. Secondly, legal documents regarding the observed transport activity and forestry laws are accessed from SUMAL. Lastly, another YOLOv8 model is used to identify singular logs in a truck, also calculating the approximate volume of wood in order to detect discrepancies. All the steps mentioned happen with the help of a mobile application with real-time processing, but without relying on external APIs or cloud infrastructure. Furthermore, the application also allows for manual confirmation of the predicted results in order to provide the most accurate results [16].

Although existing projects and initiatives help to solve parts of the problem, each has clear limitations. SUMAL 1.0 and “Inspectorul Pădurii” depend on manual updates and do not provide automated features, making timely intervention difficult. SUMAL 2.0 includes GPS tracking, but the workflow is still very much tied to administrative manual checks. Acoustic IoT projects excel at identifying the sounds of trees being cut down, but cannot directly check for individual compliance. XyloTron targets wood structure identification in supply chains, but cannot be used to detect unauthorized events as they occur. PatrolVision and Forest Guard demonstrate the feasibility of plate recognition, but they are not designed as persistent edge sensors with alert abilities. In short, none of these methods provide a fully automatic and field-deployable pipeline for detecting logging trucks, verifying authorization in real time, and issuing alerts without human initiation.

## 2. Materials and Methods

### 2.1. Design

The proposed solution for the problem of illegal deforestation is TreeHelper, an edge–IoT system for authorization and monitoring of wood transport. The system is made up of two components: a web platform and an edge AI monitoring device. Figure 1 shows a visual representation of the system architecture.

The website provides a seamless and easy way for individuals and companies in the forestry sector to request authorizations for the transport of timber, while giving authorities all the relevant information for well-informed decision-making regarding this matter; it also allows for the possibility of having a comprehensive overview of timber transports within specific administrative jurisdictions. React has been used for the frontend, Spring Boot for the backend and PostgreSQL for the relational database. A Cloudflare R2 bucket (Cloudflare, Inc., San Francisco, CA, USA) is used to securely store personal identification document images.

Users can register by providing all the required personal details, afterwards being allowed to log in via email and password. Users can make new transport authorization requests by inputting data regarding the specific transport, including the license plate number of the used vehicle. Authorities have role-specific accounts based on the county in which they act and are given a dashboard with all the wood transport authorization requests in that particular county. They are able to view which requests await confirmation and deny or approve these, with users receiving real-time updates.

The edge device is built using a Raspberry Pi 4 Model B (Raspberry Pi Ltd., Cambridge, UK), a Raspberry Pi Camera Module 3 (Raspberry Pi Ltd., Cambridge, UK), a Google Coral USB Accelerator (Google LLC, Mountain View, CA, USA) and a Waveshare SIM7600E-H 4G HAT (Waveshare Electronics, Shenzhen, China). The camera operates continuously, capturing a live feed that is processed in real time by a machine learning model trained to detect logging trucks. The USB accelerator is used in order to accelerate the inference process of the model, providing more efficiency.

Furthermore, the license plate number is extracted from the frames and is sent to the Snapshot Cloud OCR Service through the API. Afterwards, it is sent to the central server via a 4G connection provided by the SIM7600E-H (SIMCom Wireless Solutions Co., Ltd., Shanghai, China). If the license plate number is not found in the database as authorized, an alert is triggered and the relevant authorities receive an SMS notification with details, including the license plate number and GPS coordinates, so that they can make a timely intervention in response to unauthorized timber transport.

**Figure 2:**
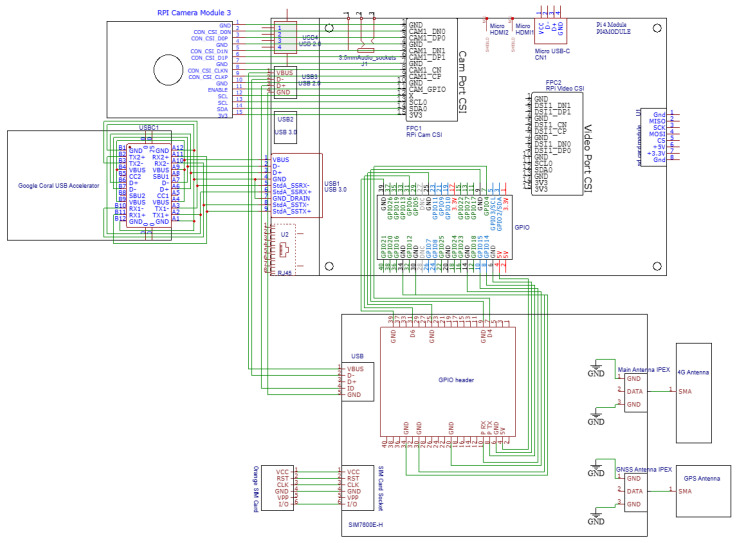
Hardware architecture.

The edge AI device of TreeHelper integrates components designed for real-time image processing, acceleration of inference, communication and geo-location, all centered around the Raspberry Pi 4 Model B. The hardware architecture is illustrated in Figure 2, with the use of EasyEDA software v6.5.48.

The Raspberry Pi Camera Module 3 connects to the Raspberry Pi via the CSI (Camera Serial Interface) port, in order to capture the live video feed. The video stream is analyzed by a machine learning model running on the edge device. In order to facilitate optimal inference speed, a Google Coral USB Accelerator is used. It is connected through a USB 3.0 interface (USB Type C on the Google Coral side, USB 3.0 on the Raspberry Pi side), allowing fast data transfer between the Raspberry Pi and the Edge TPU (Tensor Processing Unit) of the accelerator.

In the neural network, there are many interconnected nodes performing a multiply-accumulate (MAC) operation followed by an activation function. The very large number of neurons, represented by MAC cells, makes the task computationally intensive, as CPUs are very inefficient because of their sequential execution. GPUs offer parallel processing and are great for the job, but TPUs take are even better, as they hard-wire these operations in silicon. The Edge TPU uses systolic arrays, parallelized grids of MAC cells that pass intermediate results between each other in a rhythmic manner. Pipelining and buffering of intermediate results ensure the systolic array can accept new inputs every few nanoseconds, reaching very high levels of parallelism and low latency.

The core part of the TPU is the MXU (Matrix Multiplication Unit), which applies the principles mentioned previously, processing tens of thousands of matrix operations in a single clock cycle.

In order to facilitate network connectivity and positioning, the edge device uses a Waveshare SIM7600E-H 4G HAT (SIMCom Wireless Solutions Co., Ltd., Shanghai, China) that is connected to the Raspberry Pi through a 40-pin GPIO header (including the UART interface), with additional communication being possible through USB. This module provides both 4G connectivity and GNSS positioning (it supports GPS, GLONASS and BeiDou), thus allowing the system to transmit data and SMS alerts over mobile networks while also obtaining precise location coordinates.

The serial UART connection is made up of 2 pins, P TX and P RX, each corresponding to the Raspberry Pi’s counterpart, allowing for transmission and receiving of data. The power supply uses the 5V pin and several GND pins from the Pi. There also are two GPIO control lines, allowing the Pi be in control if the SIM7600E-H is in flight mode (preventing wireless transmission) or if it is powered on or off. For the device to have a cellular connection, a SIM card is required. The SIM card enables access to services provided by the mobile operator and allows for secure, encrypted data transmission. TreeHelper uses an Orange Romania PrePay SIM card. The connection is performed through a dedicated serial interface with six signal lines.

The SIM7600E-H is connected to a main antenna (stable 4G connectivity) and a GNSS antenna with the help of the IPEX (Micro Coaxial Connector) ports on the board, their main function being to provide high-frequency and high-speed signal transmission. On the antenna side, the connector is SMA (SubMiniature version A), a large and robust RF connector used for external antennas that provides a secure and highly reliable connection even in harsh environments.

**Figure 3:**
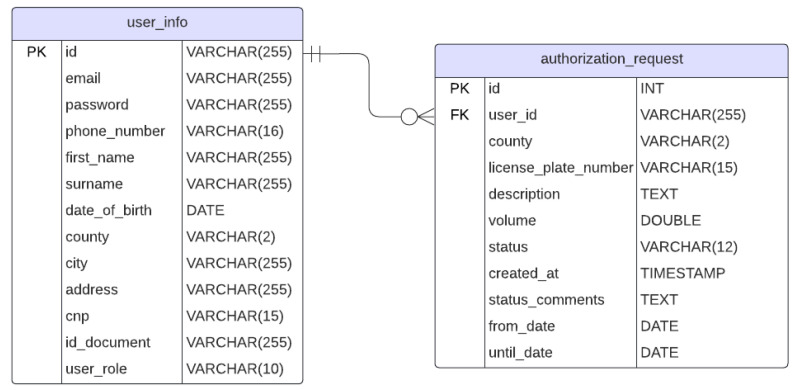
ERD of the database.

The entity relationship of the used database is shown in Figure 3. It gives an oversight over how information is stored and accessed. A more detailed website implementation description is beyond the scope of this paper. The focus will be on the monitoring system.

The use case is checking if the a license plate number is authorized, the actors are the monitoring device (primary) and the cloud ALPR service (secondary), with the main usage scenario as follows:

1. The monitoring device receives input, either a live camera feed, a video or an image.
2. The monitoring device obtains its GPS coordinates.
3. The monitoring device detects if a logging truck is in a frame.
4. If a truck is detected, the frame is captured.
5. The system sends the captured frame to the Cloud ALPR service.
6. The Cloud ALPR service extracts the license plate number as a string.
7. The monitoring device receives the license plate number.
8. The monitoring device accesses a website endpoint to check if the license plate number corresponds to a granted authorization.

Extensions:

1. If step 8 responds with a negative answer:
2. 8a. The monitoring device sends an SMS to the authorities.

### 2.2. Implementation-Machine Learning Model

The machine learning model used is YOLOv11n. The model fits this use case perfectly as it is new and performant; however, being the nano version, it is not too computationally heavy. It has been trained on a custom dataset.

The custom dataset used is derived from a Roboflow Universe dataset named “expanded-logging-trucks”, uploaded by user “loggingtruck” [26]. It consists of 1752 labeled images of logging trucks. The images are in color and the logging trucks are observed in the wild. Scenes include rural areas, forest-filled areas, plains, tracks and roads, with a multitude of backgrounds. The viewpoints include frontal, rear, and three-quarter angles, and the set covers a large range of different truck configurations transporting different kinds of timber. Visual conditions are varied, with different lighting conditions being observed in the images. Occlusions are also present, either because of foliage or other objects.

With the help of Roboflow tools, the initial dataset has been forked, allowing for different export options. The following dataset split has been chosen:

- Train Set: 1052 images (60%)
  - This portion is used directly during model training. The model learns patterns from these images, identifying trucks, understanding bounding boxes and associating visual features with logging trucks.
- Validation Set: 350 images (20%)
  - These are used during training but not for learning. They help tune hyper-parameters and monitor performance after each epoch. Loss, precision and other metrics are calculated on this set in order to detect overfitting and underfitting.
- Test Set: 350 images (20%)
  - This set is used only after training is completed. It evaluates the model’s generalization, i.e., how well it performs on unseen data.

This split is common and provides sufficient data for effective model training while also ensuring balance.

In order to train the YOLOv11n model, the “ultralytics” Python 3.13.3 package has been used. It provides a high-level API for working with YOLO models and allows for both CLI and Python code usage.

Firstly, the dataset is exported from Roboflow in YOLOv11 format. The data.yaml file contains information about the files—where the images are stored, the number and names of classes that the objects can be detected as and Roboflow-specific details about the dataset. The three sets are stored in separate folders named accordingly, and each contains another two folders: one with images and one with labels.

Training is initiated with the following parameters:

- data = .../data.yaml – path to the dataset configuration file.
- model = yolo11n.pt – specifies the usage of the YOLOv11n model.
- epochs = 100 – number of training epochs.
- imgsz = 640 – resizes all images to 640 × 640 pixels for uniform input size.

For this use case, we consider YOLOv11n a very good choice among compact detectors. Relative to earlier YOLO nanos (for example YOLOv5n or YOLOv8n), it maintains the favorable speed and accuracy characteristics of modern YOLO backbones, while remaining exportable to TFLite for Edge-TPU acceleration. Alternatives optimized for TFLite, such as SSD-MobileNetV2 and EfficientDet-Lite0/1, compile cleanly and run very fast on Edge-TPU, but they are behind modern YOLO nanos in terms of performance, especially regarding localization of objects under motion blur and occlusions, leading to possible misses or misinterpreted frames. Larger models, like YOLO s, m, l or x, can offer the highest accuracy on desktop computers, but they are, for the most part, not suitable for edge deployment because of the resources they require.

We used Ultralytics’ optimizer = auto with an initial learning rate lr0 = 0.01. During training, the learning rate was decayed by the library’s default scheduler to a final value of lr0 × lrf, with lrf = 0.01, (i.e., 10^−4^), thus providing fast early progress and stable convergence.

Once this command is executed, Ultralytics loads the architecture and initializes weights. The training loop begins and iterates across the training and validation sets for 100 epochs, and at each step, the model learns to predict bounding boxes for logging trucks. Metrics such as loss, precision, recall and mAP are logged automatically. The output contains the best.pt model, which can be used as the ML model in the application.

Initially, the Ultralyitics YOLO models are in PyTorch v2.7.0 format. The model inference is performed on a Raspberry Pi 4 Model B connected to a Google Coral USB Accelerator, which used for accelerated inference. Thus, the optimal format for the model is TensorFlow Lite for Edge TPU.

Ultralytics allows for the export to happen through a CLI command with the mention of the format; based on this, the background processes are different. In this case, the format is “edgetpu”, and an image size of 320 × 320 is chosen. The command ensures both the conversion and the compilation of the TFLite Edge TPU model, allowing for further minimal usage of power with fast performance inference.

A critical step for improving the efficiency and reducing the size of the model is quantization. Post-Training Quantization (PTQ) is applied to convert the model weights and activations from 32-bit floating-point numbers to 8-bit integers, drastically reducing the size. The quantization is handled by the Ultralytics export.

Lastly, the model is compiled for Edge TPU using a dedicated compiler, optimizing operations for running on the TPU. After compilation, the model is ready to be used on the edge device, but not all operations are supported by Edge TPU. So, the unsupported operations are offloaded to the CPU of the Raspberry Pi; however, in this model’s case, the majority of the operations are successfully mapped. The Edge TPU Compiler outputs a log with details about the mapping of operations. It is shown in Table 1.

### 2.3. Implementation—Raspberry Pi Software

The software running on the edge device, more specifically on the Raspberry Pi, is written in Python and follows object-oriented programming. Its main goal is to detect logging trucks, extract their license plate numbers, check if they are linked to an authorized transport and alert the authorities if they are not. It is structured in a modular manner so that components can easily be interconnected, maintained or modified if needed. A YOLOv11 model accelerated by the Google Coral USB Accelerator is used to perform object detection on images, videos or a live camera feed.

**Figure 4:**
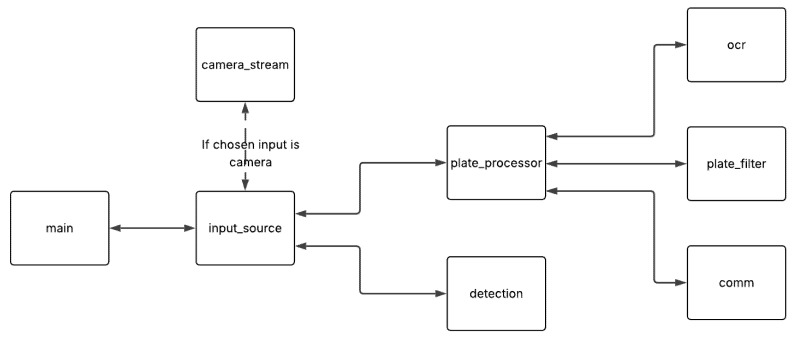
RPI code structure.

The main control flow is defined in main.py, which begins execution based on the type of input using input_source.py. The detection.py module handles detection by preprocessing input frames, running the model inference and postprocessing the outputs, helping in the final drawn position of bounding boxes. If a truck is detected, plate_processor.py coordinates the pipeline for license plate recognition using ocr.py for communication with the cloud ALPR service, buffering, reliability, deduplication and verification with plate_filter.py and for communication with the backend and authorities through comm.py. This module, comm.py, communicates through SIM7600E-H 4G, sending HTTP requests to the authorization website backend and GPS coordinates and SMS alerts to the authorities when illegal logging is detected. The structure is illustrated in Figure 4.

The entrance point for the application is the main.py script, as it starts the whole pipeline. It initializes the detector module with the previously trained model and selects one of the three input modes—image, video or live—delegating control to the corresponding function from the input_source.py file. The “live” mode has a particularity, an instance of PiCameraStream being initialized with the input dimensions defined by the YOLO model. This object continuously captures frames.

The detector class is designed specifically for quantized YOLOv11 models compiled for Edge TPU. It manages the full inference pipeline. Firstly, at instantiation, it loads the model using tflite_runtime.Interpreter. Then, the load_delegate(‘libedgetpu.so.1’) function call enables the offloading of supported operations on the Edge TPU, allowing hardware acceleration. Once this is all loaded, allocate_tensors() is called to prepare the internal buffers. Input and output details are retrieved to determine input shape, data type and quantization parameters.

Before inference, preprocessing is performed. If the model was trained and exported using uint8 quantization, the frame is simply casted into uint8 and the dimensions are expanded to include a batch size. Here, no scaling is needed, as pixel values are already in the expected [0, 255]. In contrast, if the model was quantized using int8 (this trained model’s scenario), it triggers affine quantization for the frames, with the parameters being retrieved from the model. If the model expects any other data type, an error is thrown. The output shape of this function and the input shape of the model is [1, height, width, 3].

The detection function firstly ensures that the input image has the correct spatial dimensions and resizes using cv2.resize() if needed. In our case, the input size will be 320. Then, the preprocess() function described before is called and the data is loaded into the interpreter, so that inference can start, by calling self.interpreter.invoke(). Finally, the output data is received. The prediction tensor contains bounding box coordinates, objectness scores and class confidence. Dequantization is performed through a reversal of the process described before; float32 values are again reached.

Finally, postprocessing is performed. The postprocess() function takes the raw output tensor from the model and converts it into final predictions using a confidence threshold and NMS (Non-Maximum Suppression). The output is extracted from what the model returns:

- The first four rows represent the bounding box: [x_center, y_center, width, height].
- The fifth row contains confidence scores for each prediction.

Afterwards, the confidence threshold is applied, filtering out the results that do not reach the chosen score (in this case, 0.5). The boxes are converted from center format to corner format ([x1, y1, x2, y2]) through simple geometry computations, after which they are scaled to the original input size. NMS removes overlapping boxes for the same object by keeping only the best-scoring box in a region, based on IoU. The default threshold for IoU is 0.5. Finally, a list of all the surviving boxes is returned in the format [x1, y1, x2, y2, confidence].

As mentioned before, in the case of a live camera feed, a PiCameraStream object is used. This class uses the PiCamera2 library to instantiate such an object, which sets the resolution to the shape of the model and ensures the image is in RGB888 (8 pixels for each channel) so that it is OpenCV compatible. The “preview” configuration setting is chosen, as it is recommended for real-time streaming. Finally, the camera is started and a 1 s window is set up to allow it to warm up. The get_frame() function captures and returns a single frame as a NumPy array, and “stop()” stops the camera stream and releases hardware resources.

The input_source.py module defines how detection is applied to the different kinds of input sources: images, video files or live camera feeds. In each case, the instantiated detector object is used for inference and a PlateProcessor object is used to handle confirmed logging truck detections. The draw_detections() function is used to draw the bounding boxes and confidence scores and to scale box coordinates back to original image size.

The PlateProcessor class is activated by detections and becomes the core logic that decides if an SMS alert has to be sent to the authorities. It combines OCR, filtering and telecommunication. The constructor of this class receives a sim module, a cooldown time period and a value that dictates the minimum number of confirmations needed to actually take a license plate into consideration (used in the plate filter). The attributes of the class are the plate filter, the sim module, the last time a license plate was sent to the OCR API, the cooldown and a flag that indicates if the SIM module is on or not.

To make sure the SIM7600 is initialized and ready to send SMS messages with GPS coordinates, the ensure_sim_ready(self) method is used. If it is not, the initialization process begins. The should_process(self) function prevents repeated SMS spam by enforcing a delay. The shutdown(self) method stops the SIM7600 and marks this.

The main method of the class is process_frame(). If the SIM module is ready and the should_process() function gives a positive return value, the processing begins:

1. The frame is saved temporarily as an image file.
2. The image is sent to the OCR API (with a method imported from ocr.py).
3. If a license plate number is returned, it is sent to the plate filter.
4. If the plate filter confirms everything is ok and verifies the plate, it is
5. taken by the SIM module code (from comm.py) for checking authorization.
6. If the plate is not authorized, the SIM is called in order to send the alert SMS to the authorities (also containing the location coordinates).

Automatic license plate number recognition is handled by the ocr.py module. It has only one function, send_to_plate_api(), which is responsible for performing this process by using the Plate Recognizer Snapshot API, a cloud-based OCR service. The function opens an image as binary, builds the payload (“files” contains the image file, while “data” contains the camera_id and region) and makes the API call a POST request to the provided URL, with the personal authorization token included in the header. If the call is successful, the plate is extracted, converted to uppercase and returned. If no plates are detected, “none” is returned.

A key role in ensuring reliable and non-redundant detection of license plates in the system is held by the PlateFilter class. It is designed to confirm detections only after repeated recognition (number defined by the “min_confirmations” parameter) and to avoid sending multiple alerts for the same license plate within a short time window (value defined by the “dedup_window” parameter. The add(self, plate) method is called every time a plate is recognized from a frame. If a plate is found, the buffer count for that specific string increases. If the confirmation threshold is met (in this application, the threshold is set to three), a deduplication check follows. If the plate was not verified or the last verification was longer than the deduplication window (60 s for this specific application), it is considered confirmed and returned. In this case, the buffer is also reset. Thus, this class ensures avoidance of false positives and repeated alerts for the same vehicle.

The communication backbone for the Edge IoT device comes from the comm.py script: the SIM7600 class. It controls the power and configuration of the 4G module with the same name, obtains the GPS location of the device, handles sending SMS messages and accesses a backend endpoint to check plate authorization status. The communication works with the help of AT commands and instruction to control modems and operates independently of Wi-Fi, as it uses SIM card mobile data.

The “init” method sets up serial communication on port ttyS0, sets the baudrate at 115200, configures GPIO to handle the module’s power pin (number 6) and stores the PIN code of the SIM card. The power_on and power_off methods emulate a long press on the power key to turn the module on or off. This action is required at the start and ending of the communication.

Afterwards, there are four initialization methods:

- initialize_sim: Sends the PIN code of the SiIM card and checks readiness of it.
- initialize_http: Attaches mobile data service functionality and sets up the Access Point Name (net) for HTTP.
- initialize_sms: Configures SMS mode and message center.
- initialize(): Combines all of the previously mentioned methods and fetches the GPS coordinates with the specifically designed method.

In order to receive the GPS coordinates, firstly the GPS functionality of the SIM7600 is activated. Then, the GPS fix is periodically checked until the coordinates are received or until a time period of 90 s expires. The raw data is received in NMEA style (degrees and minutes), but it is converted into standard float latitude and longitude coordinates.

HTTP authorization verification is performed through check_plate_authorization(self, plate_number). It constructs the complete URL for the necessary endpoint check with the ngrok URL of the live website and the license plate number. It performs a GET request using a sequence of AT commands, and if it receives a JSON response, it is parsed accordingly. It returns true if the license plate number is associated with a GRANTED authorization in the database, false otherwise or none if the check failed.

Furthermore, every time an AT command needs to be used in the comm.py code, the “send_at” help method is called. It is a general-purpose utility for sending AT commands and waiting for the appropriate response. It returns a tuple (success: bool, response: str). It helps with the visualization of the whole communication process. For the usage of AT commands, Minicom is used. This tool is intended for Ubuntu-based systems, allowing them to send messages to phones, routers and other external devices. It is text-based and used with the serial port.

In the case of detection of an unauthorized wood transportation activity, the relevant authority will receive an alert in the form of the earlier-described SMS message. The sender number will be the phone number associated with the SIM card inserted in the 4G module.

## 3. Results

### 3.1. Machine Learning Model

**Figure 5:**
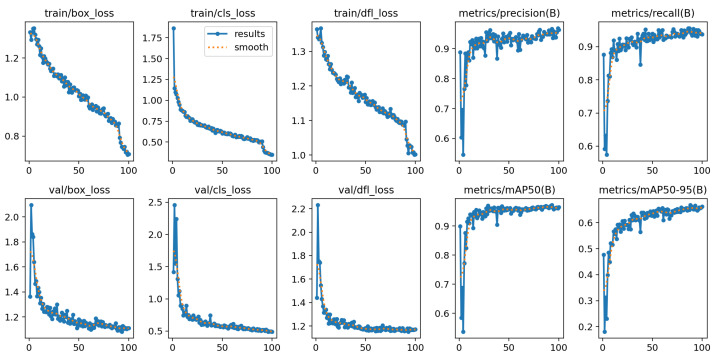
Metrics from the YOLOv11n model training process.

Figure 5 shows key metrics recorded during the machine learning training process. It includes both training and validation graphs, which help evaluate the model.

Loss Curves:

- box_loss: shows how well the model learns to predict bounding box locations.
- cls_loss: classification loss, showing the accuracy in recognizing the “logging truck” class.
- dfl_loss: distribution focal loss, used in enhancing bounding box regression precision.

All of these components show a steady decrease across training, suggesting improvement.

Performance Metrics:

- precision(B): proportion of detected objects that are actually correct.
- recall(B): proportion of actual objects that were successfully detected.
- mAP50(B): Mean Average Precision, showing a standard overall performance score. It is calculated at an IoU threshold of 0.5.
- mAP50-95(B): also Mean Average Precision, showing a standard overall performance score. It is calculated at IoU thresholds from 0.5 to 0.95.

IoU (Intersection over Union) refers to the amount of overlapping area between the ground-truth bounding box and the predicted bounding box. Both mAP metrics stabilize at high values, indicating high detection accuracy and strong bounding box precision, while precision and recall also reach values near 0.95, showing the model predicts well [27].

From the training log, the deployed YOLOv11n checkpoint achieved precision = 0.948 and recall = 0.944, with mAP50 = 0.967 and mAP50-95 = 0.668 at the best epoch, these being core metrics for live detecion accuracy.

**Figure 6:**
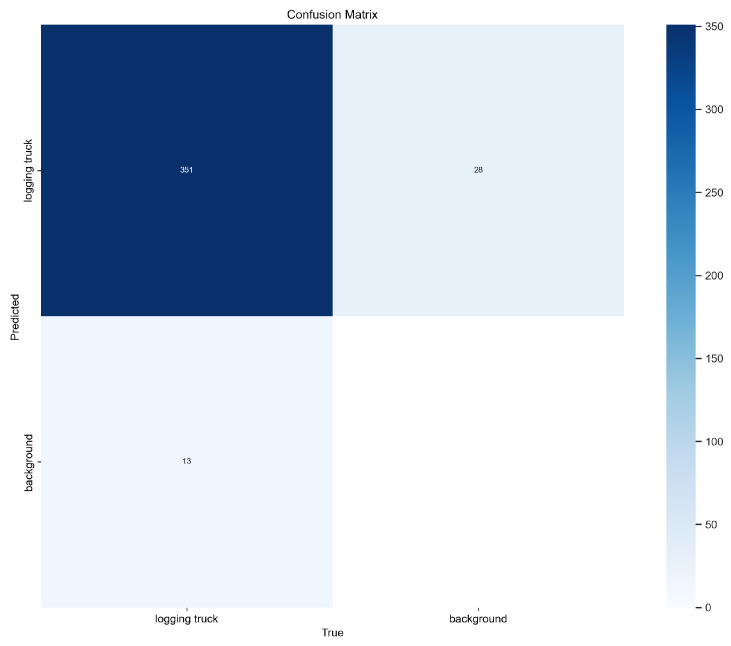
Confusion matrix.

Figure 6 shows the confusion matrix on the validation dataset with four displayed values: true positives, true negatives, false positives and false negatives. It summarizes the performance of the model, showing strong results, with most trucks being correctly classified. Not detecting a truck is considered a background detection.

**Figure 7:**
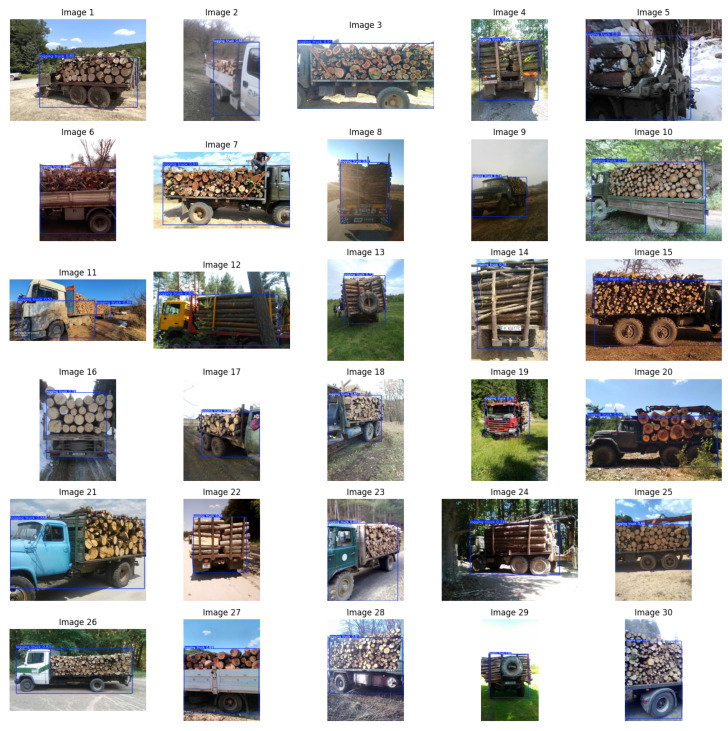
Predictions on test set images.

After training, the model is tested with the help of the test dataset. Predictions on 30 images from the set can be viewed in Figure 7. Varying accuracies can be observed, but for all of the displayed pictures, the model correctly identifies the presence of a logging truck. This outcome demonstrates the model’s ability to generalize to unseen data and confirms its ability to recognize targeted objects even under diverse conditions.

### 3.2. Live Detection Results

To evaluate the performance of the edge device, data was collected under two deployment modes: with Edge TPU acceleration and CPU-only inference. In both cases, the system was executed with identical camera input resolution and the same detection pipeline to ensure comparability. A dedicated logging script recorded performance statistics at one-second intervals into CSV files. The metric include instantaneous throughput in frames per second (FPS), the average and 95th percentile detection latency for that second and system thermals (CPU temperature). Measurements were performed both with 0 detections per second and with a varying number of detections per second. The results show that this does not have a relevant effect on the other metrics analyzed here.

**Figure 8:**
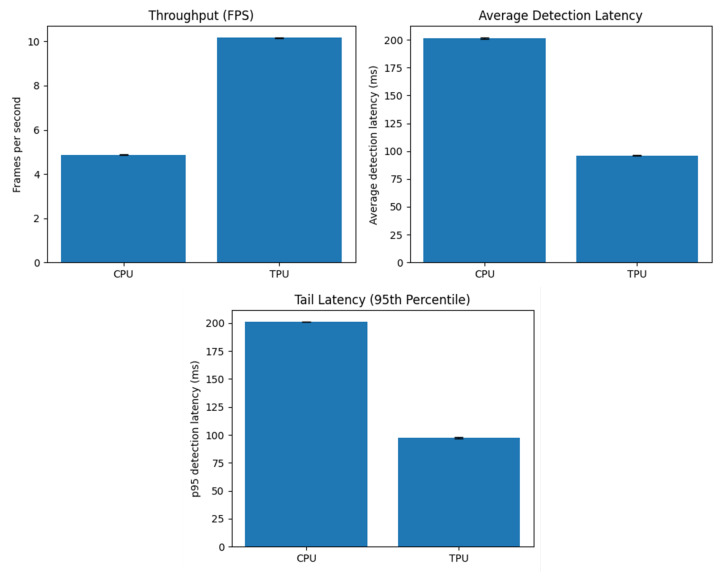
Bar plots of throughput and average/tail latency.

To visualize performance differences, we generated bar plots showing throughput and average/tail latency for the CPU vs. TPU modes (“TPU” in the figures refers to the usage of the TPU for all the compatible operations, while the CPU still does the incompatible operations, as mentioned in the previous chapter). The FPS plot shows that acceleration nearly doubles the frame processing rate, increasing the throughput from around 5 FPS on CPU to over 10 FPS with Google Coral. The latency plots highlight an equally important improvement in responsiveness: average inference time is reduced from 200 ms (CPU) to under 100 ms (TPU). The tail latency (p95) follows the same pattern, confirming that not only the mean but also the worst-case per-frame performance is significantly better with the Google Coral USB accelerator. The plots are displayed in Figure 8.

**Figure 9:**
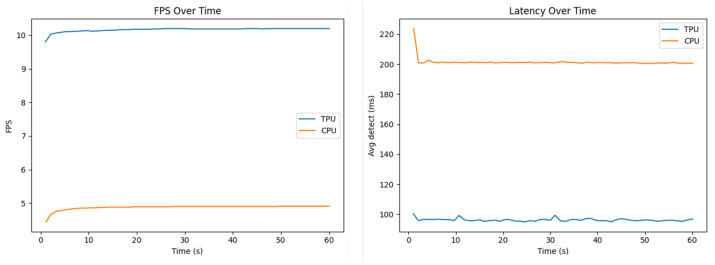
Time-series plots of FPS and latency.

Time-series plots of FPS and latency over time demonstrated the stability of both configurations. However, the TPU consistently maintains about twice the FPS and half the latency relative to CPU. These plots also confirm that neither configuration shows sudden drops or spikes, suggesting the system is thermally and computationally stable throughout the test duration. Figure 9 shows the time-series plots.

**Figure 10:**
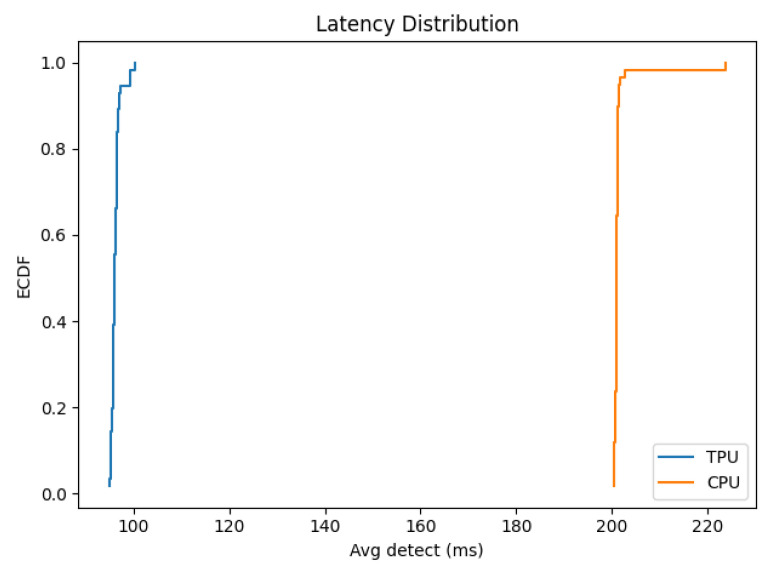
ECDF of average latency.

The empirical cumulative distribution function (ECDF) shows the full distribution of per-second average latencies. It is shown in Figure 10. The TPU curve is shifted to the left, indicating that all TPU latencies fall below the lowest CPU latencies. This plot provides a concise demonstration of how the TPU improves not just the mean but the entire latency distribution.

**Figure 11:**
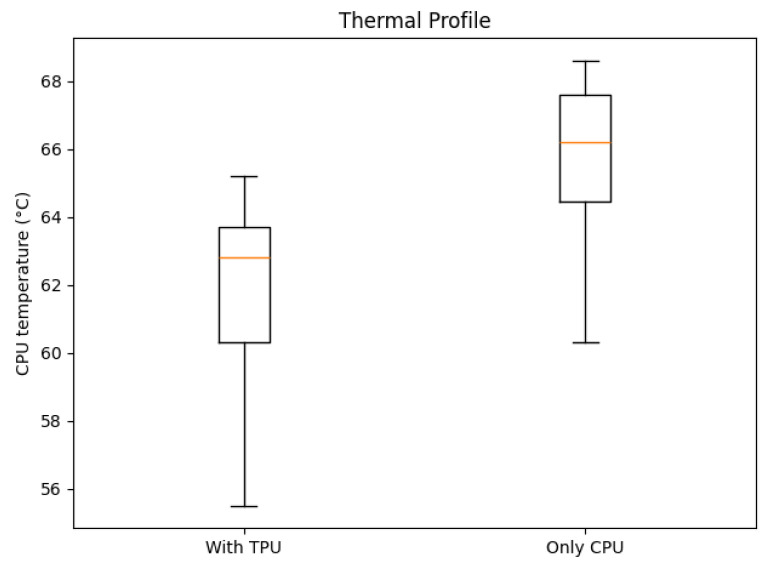
CPU temperature comparison boxplot between TPU-accelerated and CPU-only operation (no outliers detected).

The boxplot from Figure 11 compares CPU temperatures across the two runs. The run with the TPU connected shows a lower average temperature despite higher throughput. This indicates that acceleration has offloaded a significant computational burden from the CPU, reducing thermal stress.

The comparative evaluation of CPU-only and Edge TPU-accelerated inference clearly demonstrates the advantages of hardware acceleration for real-time deployment. Across all metrics, the TPU addition consistently outperformed the CPU, nearly doubling throughput and halving detection latency, while maintaining stable thermal conditions. These results confirm that hardware acceleration is essential for achieving the reliability required in continuous, real-time detection scenarios.

Furthermore, because of the design of the system and all of the experimental findings, we classify TreeHelper as having a high automation level. Detection, identification, database checks and alert transmission run continuously and end-to-end without operator input, while also happening in a short amount of time. In our experiments, although the device was not deployed for a long period of time in the environment of the use case, manual intervention was not required for the correct functioning of the pipeline. This could, of course, change in real deployment scenarios, where periodic manual checks are even recommended; however, intervention for the sake of errors would be kept at a minimum if all the relevant factors (like casing, energy and position) are taken into account. Detection of trucks from frames happens almost instantaneously. The other aspects depend on network connectivity, but our experiments have shown that communication with the cloud ALPR service also happens almost instantaneously. When the minimum number of consecutive frames with the same identified license plate is reached, there is a consistent window of under 20 s until the SMS alarm is received by the mobile phone (this timing includes the fact that messages are printed on screen during transmission to catch the exact timing and location of any errors in the pipeline).

## 4. Discussion

Our paper started as a test to see whether an unattended edge device can reliably detect illegal wood transports in real time. The results support our hypothesis that it can. With Edge-TPU acceleration, the system nearly doubled throughput and halved average detection latency, which provides evidence for truly continuous monitoring. Speed gains did not come at the expense of detection quality, as all the results show strong accuracy on previously unseen data. Together, our findings show that an inexpensive technological stack can sustain real-time performance, reaching a pragmatic point for a device that must be precise for actual useful results.

Interpreted in the context of previous efforts, TreeHelper complements the existing solutions mentioned in this paper. Existing robust administrative portals based on the information from national platforms (like SUMAL) need to be closely tied to the monitoring of transportation. Computer-vision-based detection can be enhanced using acoustics-based solutions, but we need to take into account that automated identification is not enough and the manual input variants need to exist, as they can prove to be deeply useful in particular cases that require quick intervention. Overall, all these solutions can represent the elements of a future forest protection framework in which everything is connected and communicating. This can ensure that almost no timber-related illegal activities occur, which can save whole ecosystems through the integration of procedures.

From a systems perspective, the live results suggest two practical takeaways. Firstly, partial offload to the TPU is sufficient. Even though some operators remain on the CPU after compilation, the pipeline meets real-time constraints with no real negative impact from a thermal point of view. Secondly, the alerting mechanism (plate confirmation, de-duplication and rate limiting) is as important as the detection metrics, as it allows the transition from analyzed frames to actionable and straight-forward events.

The study has some clear limitations. Although testing has been conducted extensively, weather-specific performance cannot be truly evaluated before a physical damage protection mechanism is put into place. An enclosure with heat-spreading and venting that can protect the components from wind and rain is a realistic baseline. Furthermore, energy and positioning are key elements. Exhaustive research must be conducted to select an appropriate type of energy source, and while solar panels and batteries are the first options to be taken into account, an eventual connection to an electrical network could also be a solution. Positioning needs to be performed based on an analysis of timber transport traffic in each area. After field deployment, monthly remote health checks and periodic physical inspections (including cleaning) should keep the system stable and safe for long periods of time. In our tests, the false-alert and missed events rates are close to 0; if the deployment is performed correctly and protection and maintenance are consistent, these values should not change.

In broader context, a low-cost detection device has implications beyond wood transportation: the pattern of detect, identify, verify and alert that TreeHelper facilitates could set the foundation for privacy-respecting monitoring of other transport activities.

The experiments demonstrate that real-time edge detection with reliable authorization checks is feasible on hardware that is not high-end and that our system fills a gap left by the existing administrative and acoustic systems. With more measurements, tighter integration into existing workflows and refinements for deployment, the positive results that we have obtained can be valuable for the protection of the environment.

Table 2 shows a clear progression from early systems with limited automation to increasingly intelligent solutions that leverage IoT sensors, AI and mobile connectivity. While several recent projects focus on acoustic monitoring, which is a great way of identifying illegal logging activities, this method does not give the most broad solution to the problem. TreeHelper brings an advanced mixture of technologies, elevating automation to the highest level, maintaining practicality for field deployment and bridging the gap between traditional monitoring systems and modern AI-driven solutions.

## 5. Conclusions

In conclusion, this paper describes a project that successfully demonstrates the design and implementation of an integrated system for managing wood transport authorization and monitoring, combining a modern web backend, a user-friendly frontend, cloud storage and an intelligent edge device.

A comparison of the existing solutions and TreeHelper can be seen in Table 2, emphasizing the high level of autonomy provided by the large variety of technologies utilized in the proposed system.

On the smart device side, TreeHelper makes use of a Raspberry Pi equipped with an optimized YOLOv11 model for real-time detection of logging trucks, supported by a robust OCR and communication pipeline. It demonstrates how edge computing and cloud services can work together to achieve low-latency and reliable monitoring in the field, even in environments with limited connectivity.

Overall, this paper shows how emerging technologies can be used to enhance transparency and compliance in wood transport operations, the whole forestry sector and public administration, which could contribute to ending a huge problem that the world is trying to fight—illegal deforestation.

While the system meets its intended goals, multiple directions can be followed to further improve and extend its abilities. On the machine learning side, a more advanced YOLO model, alongside an expanded dataset, would lead to greater detection accuracy in various environments, minimizing the number of false positives. Additionally, a shift from the cloud-based OCR service to a local solution could reduce latency even further.

TreeHelper could be further extended towards broader environmental and transport monitoring contexts, connecting smart vehicles with energy-efficient IoT technologies [27,28,29], while also integrating recent developments in deep-learning-based detection and physically constrained hybrid learning frameworks [30,31]. Physical Constraint-Guided (PCG) learning, which is capable of embedding real-world dynamics into model training, could enhance the robustness of future edge-AI systems being utilized for forest protection and deforestation monitoring applications.

Currently, the system needs to be connected to a constant energy source. The switch to a battery and solar panel would help with the autonomy of the device. Also, a rigid and tough casing is mandatory for actual usage on the road, in order to preemptively protect the device from human tampering or harsh weather conditions.

From a web platform perspective, the first enhancement would be advanced security measures, ensuring no vulnerabilities in either the frontend or backend. A key element would be the use of an email address for confirming credentials at the registering stage. Furthermore, there should be a focus on scalability, introducing distributed caching solutions like Redis, containerization with Docker and the use of Kubernetes for handling increased user loads efficiently. Lastly, the user experience could benefit from refinements in the frontend design, improved responsiveness on mobile devices, clearer status tracking with notifications and more complex dashboards.

Providing analytics and reporting tools for authorities could further support law compliance checks, while also generating insights into transportation trends and potential irregularities.

## Figures and Tables

**Table 1:** Edge TPU Compiler mapping log.

Operator	Count	Status
STRIDED_SLICE	12	More than one subgraph is not supported
STRIDED_SLICE	13	Mapped to Edge TPU
TRANSPOSE	2	Operation is otherwise supported, but not mapped due to some unspecified limitation
TRANSPOSE	8	More than one subgraph is not supported
TRANSPOSE	3	Mapped to Edge TPU
SOFTMAX	2	More than one subgraph is not supported
PAD	5	Mapped to Edge TPU
PAD	2	More than one subgraph is not supported
PACK	2	More than one subgraph is not supported
LOGISTIC	34	Mapped to Edge TPU
LOGISTIC	44	More than one subgraph is not supported
ADD	10	More than one subgraph is not supported
ADD	7	Mapped to Edge TPU
SPLIT	1	Mapped to Edge TPU
SPLIT	4	For example, a fully-connected or softmax layer with 2D output
FULLY_CONNECTED	4	More than one subgraph is not supported
MUL	34	Mapped to Edge TPU
MUL	46	More than one subgraph is not supported
CONV_2D	46	More than one subgraph is not supported
CONV_2D	163	Mapped to Edge TPU
DEPTHWISE_CONV_2D	6	More than one subgraph is not supported
RESIZE_NEAREST_NEIGHBOR	2	More than one subgraph is not supported
RESHAPE	13	More than one subgraph is not supported
RESHAPE	5	Mapped to Edge TPU
CONCATENATION	15	More than one subgraph is not supported
CONCATENATION	8	Mapped to Edge TPU
MAX_POOL_2D	3	Mapped to Edge TPU
QUANTIZE	3	More than one subgraph is not supported
SUB	3	More than one subgraph is not supported

**Table 2:** Comparison of the existing solutions and TreeHelper.

System	Technology	Automation Level
SUMAL 1.0	Desktop software	Low
Inspectorul Pădurii	Web and mobile application	Low
SUMAL 2.0	Web, mobile, and GPS system	Medium
Vodafone Smart Forest	Acoustic IoT and AI	High
Rainforest Connection (RFCx)	Acoustic IoT and AI	High
GreenSoal	Acoustic IoT and AI	High
XyloTron	AI wood structure analysis	Medium
PatrolVision	YOLO, OCR	Medium
Forest Guard	YOLO, OCR, mobile application	Medium
TreeHelper	YOLO, OCR, web application, 4G communication, GPS	High

## Data Availability

The original contributions presented in this study are included in the article. Further inquiries can be directed to the corresponding author.
